# Knowledge, attitudes, and behaviors toward fertility preservation in patients with breast cancer: A cross-sectional survey of physicians

**DOI:** 10.3389/fonc.2023.1109694

**Published:** 2023-01-23

**Authors:** Soo Yeon Baek, Kyung-Hun Lee, Sung-Bae Kim, Henry Gomez, Tatiana Vidaurre, Yeon Hee Park, Hee Kyung Ahn, Yoo Seok Kim, In Hae Park, Sung Gwe Ahn, Jeeyeon Lee, Jae Ho Jeong, Seonok Kim, Hee Jeong Kim

**Affiliations:** ^1^ Department of Surgery, Ajou University School of Medicine, Suwon, Republic of Korea; ^2^ Department of Internal Medicine, Seoul National University Hospital, Seoul, Republic of Korea; ^3^ Cancer Research Institute, Seoul National University, Seoul, Republic of Korea; ^4^ Department of Oncology, Asan Medical Center, University of Ulsan College of Medicine, Seoul, Republic of Korea; ^5^ Department of Medical Oncology, Instituto Nacional de Enfermedades Neoplasicas, Lima, Peru; ^6^ Division of Hematology-Oncology, Department of Medicine, Samsung Medical Center, Sungkyunkwan University School of Medicine, Seoul, Republic of Korea; ^7^ Division of Medical Oncology, Department of Internal Medicine, Gachon University Gil Medical Center, Incheon, Republic of Korea; ^8^ Department of Surgery, Chosun University College of Medicine, Chosun University Hospital, Gwangju, Republic of Korea; ^9^ Division of Hematology/Oncology, Department of Internal Medicine, Korea University College of Medicine, Guro Hospital, Seoul, Republic of Korea; ^10^ Department of Surgery, Gangnam Severance Hospital, Yonsei University College of Medicine, Seoul, Republic of Korea; ^11^ Department of Surgery, Kyungpook National University Chilgok Hospital, School of Medicine, Kyungpook National University, Daegu, Republic of Korea; ^12^ Department of Clinical Epidemiology and Biostatistics, Asan Medical Center, Seoul, Republic of Korea; ^13^ Division of Breast Surgery, Department of Surgery, University of Ulsan College of Medicine, Asan Medical Center, Seoul, Republic of Korea

**Keywords:** breast cancer, young age, fertility preservation, consultation and referral, survivorship

## Abstract

**Background:**

Fertility is an important issue for young women with breast cancer, but studies about physicians’ knowledge, attitudes, and practices toward fertility preservation (FP) are largely based on Western populations and do not reflect recent international guidelines for FP. In this international study, we aimed to assess the knowledge, attitudes, and practices of physicians from South Korea, other Asian countries, and Latin America toward FP in young women with breast cancer, and identify the related barriers.

**Methods:**

The survey was conducted anonymously among physicians from South Korea, other Asian countries, and Latin America involved in breast cancer care between November 2020 and July 2021. Topics included knowledge, attitudes, and perceptions toward FP; practice behaviors; barriers; and participant demographics. We grouped related questions around two main themes—discussion with patients about FP, and consultation and referral to a reproductive endocrinologist. We analyzed the relationships between main questions and other survey items.

**Results:**

A total of 151 physicians completed the survey. Most participants’ overall knowledge about FP was good. More than half of the participants answered that they discussed FP with their patients in most cases, but that personnel to facilitate discussions about FP and the provision of educational materials were limited. A major barrier was time constraints in the clinic (52.6%). Discussion, consultations, and referrals were more likely to be performed by surgeons who primarily treated patients with operable breast cancer (FP discussion odds ratio [OR]: 2.90; 95% confidence interval [CI]: 1.24–6.79; FP consultation and referral OR: 2.98; 95% CI: 1.14–7.74). Participants’ knowledge and attitudes about FP were significantly associated with discussion, consultations, and referrals.

**Conclusion:**

Physicians from South Korea, other Asian countries, and Latin America are knowledgeable about FP and most perform practice behaviors toward FP well. Physicians’ knowledge and favorable attitudes are significantly related to discussion with patients, as well as consultation with and referral to reproductive endocrinologists. However, there are also barriers, such as limitations to human resources and materials, suggesting a need for a systematic approach to improve FP for young women with breast cancer.

## Introduction

Breast cancer is the most common cancer and a leading cause of death among women (1). The incidence of invasive breast cancer in women of reproductive age is 73.2 cases per 100,000, which far exceeds the incidence of other cancers in young adults from 2011 to 2015 (2). However, advances in treatment have led to gradual improvements in survival rates over the past 20 years. As long-term survival is expected, survivorship issues, such as fertility and quality of life, are becoming more important (3, 4).

Despite the improvements in survival rates, women with breast cancer have a lower birth rate than those with other cancers, as well as the general population (5). Younger patients with breast cancer often have more biologically aggressive tumor characteristics than older patients, and tend to receive more chemotherapy, which can lead to premature ovarian failure (6). Chemotherapy-induced amenorrhea may be reversible, but in some cases, ovarian function is not restored for years (7). Furthermore, the ovarian reserve is reduced after the completion of chemotherapy (8). In addition, for patients with hormone receptor-positive tumors, standard endocrine therapy includes tamoxifen for at least five or even ten years, with or without two to five years of ovarian suppression (9). Owing to the long duration of treatment, patients age after treatment, and their fecundity decrease accordingly (10).

Fertility loss causes considerable distress and results in various psychosocial problems among cancer survivors (4). In particular, younger patients with breast cancer are more vulnerable to distress than older ones (11, 12). More than 50% of patients of childbearing age with breast cancer have concerns about treatment-related early menopause or infertility (13). These fertility concerns can also lead to problems with treatment adherence (14), and non-adherence is related to low survival rates (15). Therefore, counseling for fertility preservation (FP) is recommended to reduce dissatisfaction and concerns regarding loss of fertility in young patients (16).

Current international guidelines recommend advising patients about the potential risk to fertility as early as possible before treatment starts, to allow for the full range of options for FP (17). Several factors have been identified that can influence FP counseling or procedures for patients with cancer (18–21). Regarding physician factors, several survey studies have been conducted about FP in patients with cancer (19–25); most have focused on Western populations. However, there are differences in the patterns of breast cancer in Western and Asian countries, with a lower peak age in Asian countries (26). In Asia, more than 40% of patients with breast cancer are diagnosed under the age of 50, compared with about 20% in Western countries (27). Nevertheless, in a study of FP experts in Asian countries, it was reported that low recognition among medical staff is one of the major issues hampering FP for childhood and adolescent patients (28). In addition, previous studies on Asian physicians treating breast cancer are single-nation studies conducted prior to the American Society of Clinical Oncology guidelines update in 2016 (22, 25).

Therefore, we conducted a survey study to assess the knowledge, attitudes, and practices of physicians toward FP in young women with breast cancer, to identify barriers, and to elucidate which factors are associated with FP discussion and referral to infertility specialists among physicians in South Korea, other Asian countries, and Latin America.

## Materials and methods

### Survey instrument

Based on previous studies (20, 23), we developed a questionnaire consisting of 45 questions in 6 categories: knowledge about FP, practice behaviors, barriers to FP, attitudes toward FP, perceptions on FP, and participant demographics.

Questions were answered using a five-point Likert scale, except for demographics and one question regarding knowledge about FP. For some behaviors and barriers, participants could fill out their own answers. Regarding knowledge about FP, for statements A-1 and A-4, “strongly agree” and “agree” were considered correct answers. For statement A-5, “required” was considered a correct answer (**Supplementary Table 1**). The total score was calculated by assigning one point to each correct answer. We categorized participants into knowledgeable and not knowledgeable groups based on the score distribution.

Regarding attitudes toward FP, we calculated the total score by assigning one point to the response corresponding to the positive attitude. Subsequently, participants were divided into three groups based on the score distribution. The odds were estimated with three groups, but as zero and one showed a closer effect size; they were grouped together, and two was analyzed separately. As a result, we categorized the participants into two groups: those with favorable or unfavorable attitudes. Since preimplantation genetic diagnosis is prohibited by law in South Korea, the related attitudinal survey item was not included in the analysis. Perceptions of FP were also divided into two groups based on the sum of the survey responses.

Participants’ demographics included questions on medical training and practice information. The questionnaire was developed in English, and the translated Korean version, which did not contain a nationality question, was used in the group consisting only of South Koreans.

### Procedures

The survey was conducted four times over nine months, each time with a different group. First, the survey sample included members of the Korean Breast Cancer Society (KBCS)— mainly surgeons who treat patients with breast cancer. A link to the Korean version of the survey was sent to KBCS members whose email addresses were secured in November 2020 (n = 150). Second, an English version of the survey link was distributed onsite to participants of the Asian Breast Cancer Network (ABCN) meeting held during the 2021 Global Breast Cancer Conference in Seoul, South Korea (n = 30). The ABCN meeting is a platform for the selection of joint research projects for breast cancer in the Asia-Pacific region, and for the discussion organized by the KBCS of the practical problems and solutions facing each participating country. Third, during a virtual conference about breast cancer in young women with Latin American physicians held on May 11, the survey link was released to attendees (n = about 80). Finally, a link to the Korean questionnaire was emailed to members of the Breast Committee of the Korean Cancer Study Group (n = 109), a clinical trial organization mainly composed of medical oncologists. This study was reviewed and approved by the Institutional Review Board of the Asan Medical Center (2020-1828, 2021-1463), which also waived the requirement for informed consent because of the anonymity of the survey.

### Statistical analysis

We grouped related questions around two main topics and analyzed their associations with other survey items. The first main topic was discussions about FP with patients; example items include “I discuss fertility issues with patients regardless of pathologic staging of cancer,” and “I discuss the impact of cancer treatment on future fertility with my cancer patients.” The second was consultation and referral to an infertility specialist or reproductive endocrinologist; example items include “I consult an infertility specialist or reproductive endocrinologist with questions about potential fertility issues in my patients,” and “I refer patients who have questions about fertility to an infertility specialist or reproductive endocrinologist.” Owing to the correlations between items, the sum of the items was calculated and used as a covariate (five items for physicians’ knowledge, four items for attitudes, and two items for perceptions).

Logistic regression was used to assess the association between key questions and physicians’ knowledge, attitudes, and characteristics. Odds ratios (ORs) and their 95% confidence intervals (CIs) were estimated. The candidate variables with *P* < 0.2 in univariate analysis were evaluated in multivariable analysis using backward elimination. The data of two participants who did not respond to demographic items were excluded from the univariate and multivariate analyses. *P*-values < 0.05 were considered statistically significant. All statistical analyses were performed using SPSS version 21.0 (IBM Corp., Armonk, NY, USA).

## Results

### Participant characteristics

A total of 151 physicians responded to the survey. Participants’ demographic and other characteristics are listed in **Table 1**. Of the participants, 71 (47.3%) were women. Totally, 52 (34.4%) and 97 (64.2%) of the participants specialized in oncology and surgery, respectively. A total of 64.9% of the participants were affiliated with teaching/university hospitals. Less than half of the physicians responded that they see more than 20 patients with newly diagnosed breast cancer per month. On the contrary, 26.4% responded that they see over 20 breast cancer patients every month in the 18–45 age group. One hundred and twenty-three (82.0%) physicians had children. The majority of the participants were Korean (74.7%), followed by Peruvian (9.3%) and Japanese (7.3%).

**Table 1:** Participants’ characteristics (N = 151).

Characteristic	Values (%)
Sex	
Male	79 (52.7)
Female	71 (47.3)
Unknown	1
Religious background	
Catholic	54 (37.0)
Christian	34 (23.3)
Buddhist	13 (8.9)
Atheist	40 (27.4)
Others	5 (3.4)
Unknown	5
Year of graduation from medical school	
2000 or earlier	71 (49.7)
2001 or later	72 (50.4)
Unknown	8
Specialty	
Oncology	52 (34.4)
Surgery	97 (64.2)
Others	2 (1.3)
Type of affiliation	
Teaching/university affiliated	98 (64.9)
National cancer institute	26 (17.2)
Private office	22 (14.6)
Others	5 (3.3)
Primary practice location	
Capital city	107 (70.9)
Provincial city	29 (19.2)
Provincial town	14 (9.3)
Rural area	1 (0.7)
Practice arrangement	
Resident	6 (4.0)
Fellow	4 (2.7)
Professor	111 (73.5)
Employee	18 (11.9)
Owner	9 (6.0)
Others	3 (2.0)
No. of physicians working together	
≤5	89 (62.7)
>5	53 (37.3)
Unknown	9
No. of newly diagnosed patients with breast cancer seen per month	
≤20	74 (51.4)
>20	70 (48.6)
Unknown	7
No. of patients with breast cancer aged 18–45 seen per month	
≤20	106 (73.6)
>20	38 (26.4)
Unknown	7
No. of children	
0	27 (18.0)
1	39 (26.0)
2	61 (40.7)
3	23 (15.3)
Unknown	1
Family history of cancer	
No	45 (30.2)
Yes	104 (69.8)
Unknown	2
Nationality	
South Korea	112 (74.7)
Other Asian countries	18 (12.0)
Latin America and others	20 (13.3)
Unknown	1

### Survey results

**Figure 1:**
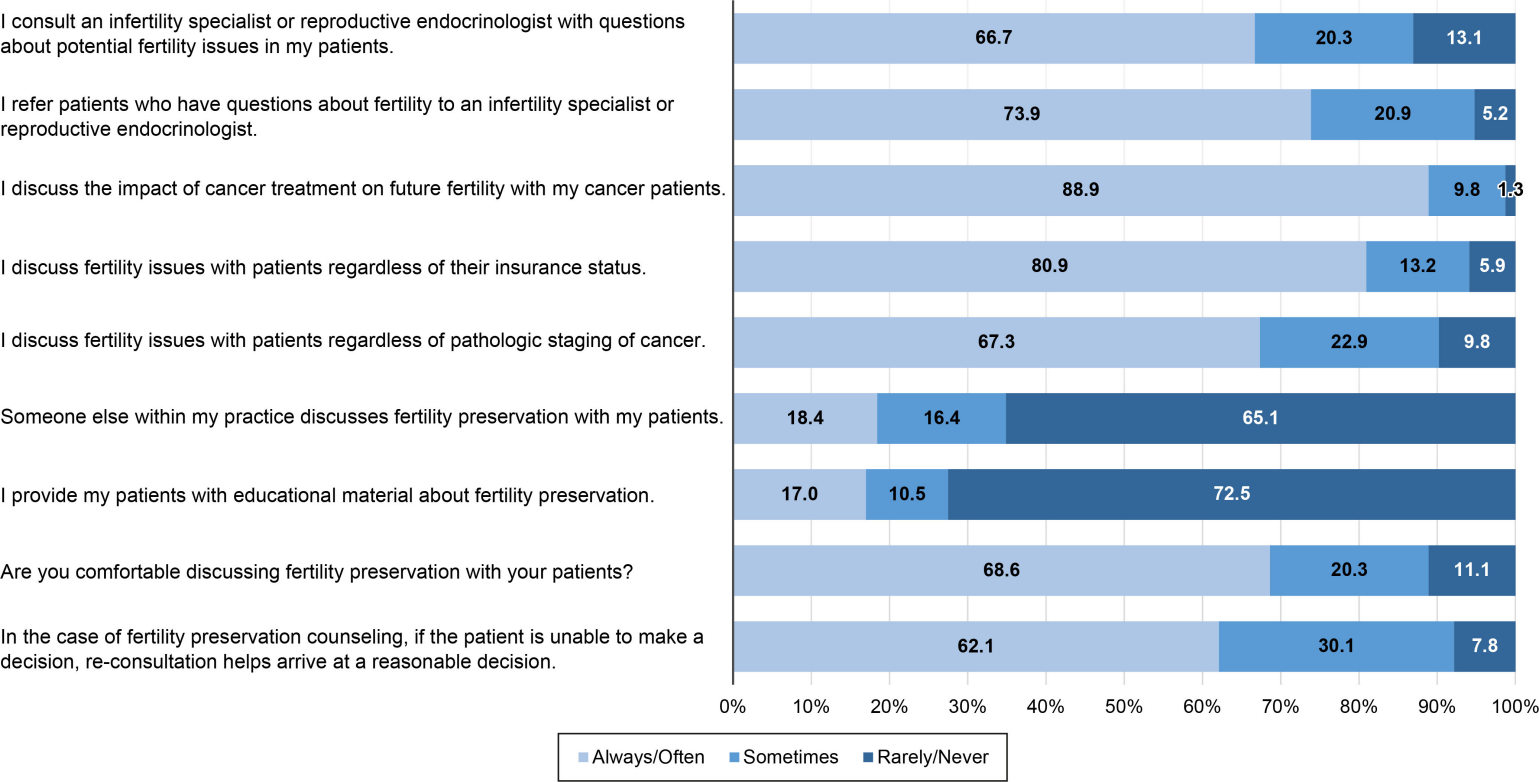
Fertility preservation practice behaviors.

The survey results are shown in the **Supplementary Tables**. Most of the participants had adequate knowledge about FP (**Supplementary Table 1**). Regarding practice behaviors, more than half of the participants always/often consulted with and referred patients with fertility issues to an infertility specialist or reproductive endocrinologist, discussed FP with their patients, and felt comfortable discussing FP with their patients. On the contrary, more than half the participants said they rarely/never provided their patients with educational materials about FP, and reported that someone else within their practice rarely/never discussed FP with their patients (**Figure 1**, **Supplementary Table 2**). The mean time spent on discussions about FP was 13.2 ± 10.4 minutes.

**Figure 2:**
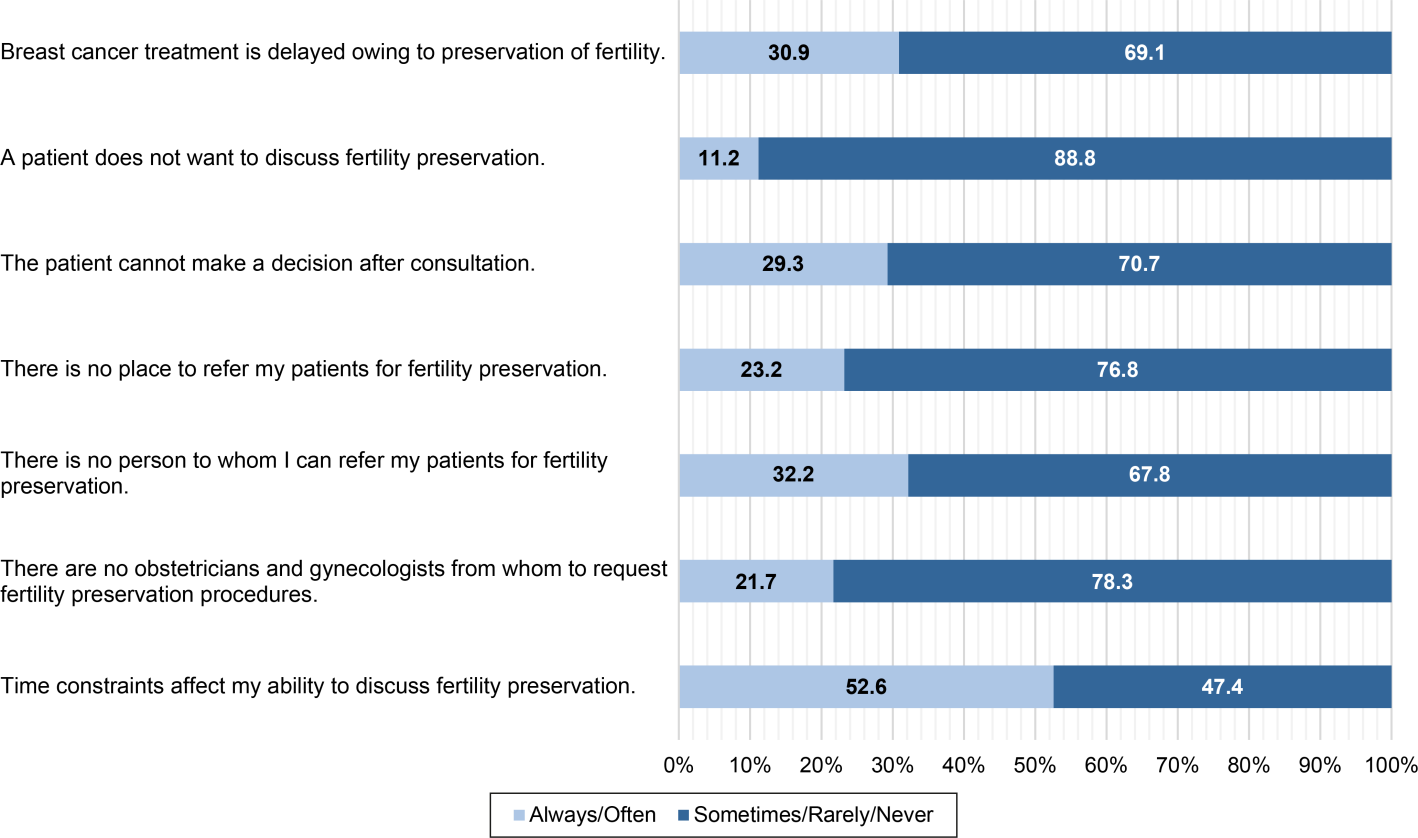
Barriers to discussing fertility issues.

In terms of barriers, the most common response was time constraints in the clinical setting (52.6%), followed by lack of human resources to whom to refer patients for FP (32.2%) (**Figure 2**, **Supplementary Table 3**). Patients’ unwillingness to discuss FP was the least common barrier (11.2%). In addition to the questionnaire items, the participants indicated other barriers to FP counseling, such as fear of recurrence and financial problems.

### Univariate analysis

In terms of participants’ characteristics, factors significantly associated with discussion were nationality (*P* < 0.001) and the number of patients with breast cancer aged 18–45 seen per month (*P* = 0.049). Factors significantly associated with consultation and referral were nationality (*P* = 0.005), number of patients with breast cancer aged 18–45 seen per month (*P* = 0.028), and family history of cancer (*P* = 0.010). For barriers, except for two items, the rarer the barrier was, the more frequent discussions with patients or consultations and referrals were performed. The group that showed favorable attitudes toward FP had more discussions, consultations, and referrals than the group with unfavorable attitudes (**Table 2**).

**Table 2:** Univariate analysis.

Variables	Discuss potential fertility issues with my patients (event = 108)			Consult with and refer patients who have questions about fertility to an infertility specialist or reproductive endocrinologist (event = 97)		
	Odds ratio	95% CI	*P*-value	Odds ratio	95% CI	*P*-value
Demographics						
Sex						
Male	1			1		
Female	1.05	0.52–2.13	0.898	1.07	0.55–2.08	0.849
Religion						
No	1			1		
Yes	1.04	0.47–2.30	0.929	1.03	0.49–2.19	0.937
Nationality						
South Korea	1		<0.001	1		0.005
Other Asian countries	Infinity		1.000	2.47	0.67–9.06	0.174
Latin America and others	0.42	0.16–1.10	0.077	0.27	0.10–0.72	0.009
Year of graduation from medical school						
2000 and before	1			1		
After 2000	0.55	0.26–1.14	0.109	1.09	0.55–2.15	0.813
Specialty						
Oncology	1			1		
Surgery and others	2.07	1.00–4.28	0.051	1.74	0.87–3.48	0.117
Primary practice location						
Urban	1		0.216	1		0.298
Suburban	0.53	0.22–1.25	0.147	0.80	0.34–1.86	0.599
Rural area	0.48	0.16–1.48	0.202	0.43	0.14–1.27	0.125
Type of affiliation						
Private office and others	1			1		
Teaching/university affiliated/National cancer institute	1.07	0.43–2.67	0.884	1.30	0.55–3.04	0.552
Practice arrangement						
Employee	1		0.338	1		0.029
Resident/Fellow/Professor	1.85	0.66–5.18	0.244	2.18	0.80–5.94	0.126
Full or part owner	1.27	0.24–6.82	0.778	1.25	0.25–6.23	0.785
Others	0.32	0.02–4.20	0.384	Infinity		1.000
No. of physicians working together						
≤5	1			1		
>5	1.27	0.59–2.76	0.546	1.09	0.53–2.23	0.810
No. of newly diagnosed patients with breast cancer seen per month						
≤20	1			1		
>20	1.72	0.82–3.60	0.148	1.90	0.95–3.81	0.068
No. of patients with breast cancer aged 18–45 seen per month						
≤20	1			1		
>20	2.63	1.01–6.88	0.049	2.66	1.11–6.35	0.028
No. of children						
0	1			1		
≥1	1.96	0.82–4.65	0.130	0.87	0.36–2.09	0.750
Family history of cancer						
No	1			1		
Yes	1.17	0.54–2.50	0.690	2.58	1.25–5.31	0.010
Fertility preservation knowledge						
Not knowledgeable	1			1		
Knowledgeable	1.88	0.91–3.90	0.090	1.61	0.80–3.23	0.178
Barriers to discussing fertility preservation						
Breast cancer treatment is delayed owing to preservation of fertility.						
Always/Often	1		0.509	1		0.316
Sometimes	1.63	0.71–3.74	0.246	1.70	0.77–3.77	0.188
Rarely/Never	1.36	0.54–3.42	0.517	1.01	0.43–2.41	0.979
A patient does not want to discuss fertility preservation.						
Always/Often	1		0.925	1		0.634
Sometimes	0.95	0.28–3.22	0.937	0.78	0.24–0.46	0.666
Rarely/Never	1.11	0.35–3.49	0.857	1.11	0.37–3.30	0.851
The patient cannot make a decision after consultation.						
Always/Often	1		0.826	1		0.049
Sometimes	0.91	0.38–2.19	0.834	1.91	0.85–4.30	0.119
Rarely/Never	1.19	0.47–3.03	0.710	2.94	1.22–7.12	0.017
There is no place to refer my patients for fertility preservation.						
Always/Often	1		0.038	1		<0.001
Sometimes	1.20	0.48–3.03	0.699	1.82	0.73–4.51	0.198
Rarely/Never	2.81	1.15–6.86	0.023	6.32	2.59–15.43	<0.001
There is no person to whom to refer my patients for fertility preservation.						
Always/Often	1		0.020	1		<0.001
Sometimes	1.49	0.60–3.75	0.393	4.38	1.70–11.27	0.002
Rarely/Never	3.21	1.38–7.51	0.007	8.21	3.47–19.41	<0.001
There are no obstetricians and gynecologists from whom to request fertility preservation procedures.						
Always/Often	1		0.009	1		<0.001
Sometimes	0.52	0.17–1.58	0.248	1.50	0.49–4.63	0.481
Rarely/Never	2.20	0.93–5.22	0.073	7.70	3.21–18.47	<0.001
Time constraints affect my ability to discuss fertility preservation.						
Always/Often	1		0.003	1		0.001
Sometimes	3.78	1.42–10.03	0.008	2.11	0.94–4.73	0.069
Rarely/Never	3.47	1.21–9.97	0.021	6.11	1.96–19.06	0.002
Fertility preservation attitudes						
Unfavorable	1			1		
Favorable	3.21	1.24–8.31	0.016	4.93	1.92–12.66	0.001
Fertility preservation perceptions						
Unfavorable	1			1		
Favorable	1.55	0.75–3.23	0.241	1.59	0.80–3.17	0.183

### Factors associated with discussions, consultations, and referrals

**Table 3** shows the multivariate analysis of key questions and other variables. Surgeons, who primarily see patients with operable breast cancer, were more likely to engage in discussions with them (OR: 2.90; 95% CI: 1.24–6.79) and consult with and refer patients who had questions about fertility to an infertility specialist or reproductive endocrinologist than medical oncologists (OR: 2.98; 95% CI: 1.14–7.74). Physicians who saw more than 20 patients aged 18–45 were more likely to discuss fertility issues with patients (OR: 3.55; 95% CI: 1.23–10.27). Physicians with a family history of cancer were more likely to consult and refer patients who had questions about fertility to an infertility specialist or reproductive endocrinologist (OR: 4.90; 95% CI: 1.69–14.21).

**Table 3:** Factors associated with discussion with patients and consultation and referral to specialists.

Variables	Discuss potential fertility issues with my patients (event = 108)			Consult with and refer patients who have questions about fertility to an infertility specialist or reproductive endocrinologist (event = 97)		
	Odds ratio	95% CI	*P*-value	Odds ratio	95% CI	*P*-value
Demographics						
Specialty						
Oncology	1			1		
Surgery and others	2.90	1.24–6.79	0.014	2.98	1.14–7.74	0.025
No. of patients with breast cancer aged 18–45 seen per month						
≤20	1					
>20	3.55	1.23–10.27	0.019			
Family history of cancer						
No				1		
Yes				4.90	1.69–14.21	0.003
Fertility preservation knowledge						
Not knowledgeable	1			1		
Knowledgeable	2.56	1.10–5.92	0.028	2.86	1.04–7.89	0.042
Barriers to discussing fertility preservation						
There is no person to whom to refer my patients for fertility preservation.						
Always/Often				1		0.011
Sometimes				5.55	1.46–21.04	0.012
Rarely/Never				4.54	1.32–15.69	0.017
There are no obstetricians and gynecologists from whom to request fertility preservation procedures.						
Always/Often	1		0.039	1		0.004
Sometimes	0.45	0.13–1.53	0.200	2.92	0.66–12.98	0.159
Rarely/Never	1.78	0.69–4.62	0.236	7.28	2.13–24.93	0.002
Time constraints affect my ability to discuss fertility preservation.						
Always/Often	1		0.021	1		0.049
Sometimes	3.74	1.29–10.80	0.015	1.89	0.65–5.51	0.246
Rarely/Never	2.44	0.78–7.56	0.123	5.97	1.29–27.69	0.023
Fertility preservation attitudes						
Unfavorable				1		
Favorable				5.38	1.58–18.39	0.007

Participants who had better knowledge engaged in more discussions (OR: 2.56; 95% CI: 1.10–5.92) and consultations and referrals (OR: 2.86; 95% CI: 1.04–7.89) compared to those who did not. Physicians with favorable attitudes toward FP engaged in more consultations and referrals compared to those with unfavorable attitudes (OR: 5.38; 95% CI: 1.58–18.39).

In terms of barriers, the availability of infertility specialists to whom to make requests regarding FP procedures was associated with discussion (*P* = 0.039) and consultation and referral (*P* = 0.004). Time constraints were associated with discussion (*P* = 0.021) and consultation and referral (*P* = 0.049). In addition, participants who did not have personnel to whom to refer patients for FP engaged in significantly less consultation and referral (*P* = 0.011).

## Discussion

Fertility information is a well-known unmet need among young adult cancer survivors (29). In addition to the lack of information, decisional conflicts about FP arise in complex decision-making processes (18). In a previous study, the prevalence of high-decisional conflict was significantly higher among participants not referred for FP counseling (30). Therefore, providing necessary information through appropriate referral to infertility specialists is important in survivorship care for patients of reproductive age.

In this study, we report the current knowledge, attitudes, and practices of physicians who treat young women with breast cancer, as well as the barriers they face in the clinical setting. We also identified factors associated with discussions with patients about FP and referral to infertility specialists. Multiple factors pertaining to participants’ characteristics have previously been reported in relation to referrals (20, 22, 25). Our study finds that specialty, number of patients with breast cancer aged 18–45 seen per month, and family history of cancer were related to discussion or consultation and referral. Quinn et al. (20) showed that the gender of oncologists is related to referral, with female oncologists making more referrals (*P* = 0.004). Shimizu et al. (22) also reported that female physicians and physicians aged under 50 are more likely to refer patients to a reproductive specialist. However, in our study, there were no significant differences between male and female physicians with regard to FP discussion and consultation and referral. These variations are likely due to differences in study populations and participants’ socio-demographics. Interestingly, in this study there was a significant difference in discussion, consultation, and referral depending on the participants’ specialty. The odds of FP discussion were about 2.90-fold higher among surgeons compared to medical oncologists, and the odds of consultation and referral to infertility specialists were 2.98-fold higher among surgeons. In South Korea, where most of the participants live, surgeons often diagnose and treat patients with operable breast cancer first, and then refer them to medical oncologists. Therefore, surgeons would have more opportunities to discuss FP with patients before treatment.

In our survey, physicians with higher knowledge scores tended to discuss more (OR: 2.56) and engage in more consultation and referral (OR: 2.86). In addition, physicians with favorable attitudes toward FP tended to consult and refer more (OR: 5.38). This result is comparable to previous studies (20, 22). On the contrary, Son et al. (25) reported that knowledge and attitudes about FP are not correlated with actual practice. Although we divided the sample into not knowledgeable and knowledgeable groups based on the response distribution, many participants responded correctly. Therefore, it is difficult to state that the not knowledgeable group actually lacked absolute knowledge about FP, and is a relative classification. Nevertheless, it is true that the group with higher knowledge scores engaged in more counseling and referral. These results suggest that more consultations and referrals will be possible if physicians’ knowledge and attitudes are improved with attention and effort.

Although our findings indicate that practices regarding FP were well performed by physicians, the provision of educational materials about FP to patients was not satisfactory. Only 17% of the participants provided educational materials about FP to patients. Sallem et al. (24) reported that 76% of oncologists responded that educational materials could be helpful in discussing fertility. We previously reported that about half of young patients with breast cancer were unaware of the effects of anticancer treatment on ovarian function and fertility, but after viewing educational videos provided by healthcare professionals, only 2% of patients answered that they had no knowledge (31). Several FP decision aids for patients with breast cancer have been developed and systemized, and their effectiveness has been proven (32, 33). Therefore, standardized educational materials and decision aids for FP should be developed and distributed to improve current practices.

This study also shows that several barriers still exist in clinical settings—and the most frequently reported barrier was time constraints. This problem has been previously reported in a South Korean study (25). In that study, patients’ lack of interest in FP was also reported as a significant barrier (25). However, the percentage of “patients did not want to discuss FP” was the lowest (11.2%) among the survey items regarding barriers in our study. Time constraints, a lack of people to consult with, and an absence of infertility specialists to request FP procedures are systematic barriers that cannot be resolved by individual physicians’ efforts. Kelvin et al. (33) reported that a hospital-level cancer and fertility program resulted in significant improvements in patient satisfaction and helpfulness of information about treatment-related fertility risks and FP options. Therefore, support and establishing programs at the hospital and national level will be needed to overcome obstacles and improve fertility-related practices.

Physician nationalities had little impact on key questions. The responses of physicians from South Korea, other Asian countries, and Latin America were mostly similar (data not shown)—with one exception. Participants from South Korea and Latin America tended to answer that time constraints affected their FP discussions “always/often” (58.9% and 55%, respectively), while only 16.7% of participants from other Asian countries responded similarly (**Supplementary Figure 1**). This is likely a selection bias regarding physicians from other Asian countries who were invited to the ABCN meeting, which is related to joint breast cancer research in the Asia-Pacific region. The number of participants from other countries was not large enough to evaluate differences among nationalities. A large multinational survey is required to reveal national differences related to FP environments and practices in the future.

This study has several limitations. First, we could not include all the relevant physicians from each country, restricting generalizability to all physicians from the studied countries or continents. Second, there may be a selection bias for physicians from other Asian countries who attended the ABCN meeting because only selected physicians from each country were invited. Third, physicians who are interested in FP are more likely to respond to the survey through e-mail or a survey link during the virtual conference. Therefore, the results that most of the practices pertaining to FP are well-performed may be biased. Finally, we did not collect data on the procedure and pathway of discussion, consultation, and referral as well as patients’ adherence to it. Further study is warranted to identify how fertility issues are discussed and through which pathways referrals and consultations are implemented in actual patient care. However, the results are meaningful in that the survey area was extended to other countries, not limited to Western countries or South Korea. In addition, this survey included physicians from a variety of specialties.

In conclusion, this survey demonstrates physicians’ current knowledge, attitudes, and practices toward FP for patients with breast cancer and reveals the obstacles to FP. Physicians’ knowledge about FP is generally high. Most of the practices pertaining to FP are well-performed among physicians in South Korea, other Asian countries, and Latin America. However, there is room for improvement regarding time constraints, a lack of personnel to consult, and poor collaboration with infertility specialists. Systematic approaches to overcome barriers are needed to improve the FP discussion and referral of patients with breast cancer.

## Data availability statement

The original contributions presented in the study are included in the article/**Supplementary Material**. Further inquiries can be directed to the corresponding author.

## Ethics statement

The studies involving human participants were reviewed and approved by Institutional Review Board of Asan Medical Center. Written informed consent for participation was not required for this study in accordance with the national legislation and the institutional requirements.

## Author contributions

Study design was contributed by HK. Data acquisition was contributed by K-HL, S-BK, HG, TV, YP, HA, YK, IP, SA, JL, JJ, and HK. Data analysis was contributed by SB and SK. Manuscript writing was contributed by SB and K-HL. All authors contributed to the article and approved the submitted version.
