# Supplementary material for: Knowledge, attitudes, and behaviors toward fertility preservation in patients with breast cancer: A cross-sectional survey of physicians
Source: Front Oncol. 2023 Jan 23;13:1109694. doi: 10.3389/fonc.2023.1109694 (PMC9899882; doi:10.3389/fonc.2023.1109694)
Supplement: Supplementary file 1 [file DataSheet_1.docx]

Supplementary Material

**Supplementary Table 1.** Fertility preservation knowledge.

|  | **Strongly agree** | **Agree** | **Neutral** | **Disagree** | **Strongly disagree** | **No opinion** |
| --- | --- | --- | --- | --- | --- | --- |
|  | **n (%)** | **n (%)** | **n (%)** | **n (%)** | **n (%)** | **n** |
| A-1. Alkylating agents have been linked to infertility in cancer patients. | 69 (45.1) | 81 (52.9) | 3 (2.0) | 0 (0.0) | 0 (0.0) | 0 |
| A-2. Some breast cancer treatments can cause early menopause in females. | 98 (64.1) | 55 (35.9) | 0 (0.0) | 0 (0.0) | 0 (0.0) | 0 |
| A-3. Oocyte preservation and embryo cryopreservation are the established methods of fertility preservation. | 63 (41.2) | 79 (51.6) | 9 (5.9) | 2 (1.3) | 0 (0.0) | 0 |
| A-4. It is recommended to use ovarian suppression injections to protect ovarian function during chemotherapy. | 56 (36.8) | 73 (48.0) | 18 (11.8) | 5 (3.3) | 0 (0.0) | 1 |
|  | **Required** | | **Not required** | | **No opinion** | |
| A-5. In the case of anti-hormonal therapy alone, consultation on preservation of fertility is required. | 131 (85.6) | | 22 (14.4) | | 0 | |

**Supplementary Table 2.** Fertility preservation practice behaviors.

|  | **Always** | **Often** | **Sometimes** | **Rarely** | **Never** | **No opinion** |
| --- | --- | --- | --- | --- | --- | --- |
|  | **n (%)** | **n (%)** | **n (%)** | **n (%)** | **n (%)** | **n** |
| B-1. I consult an infertility specialist or reproductive endocrinologist with questions about potential fertility issues in my patients. | 35 (22.9) | 67 (43.8) | 31 (20.3) | 20 (13.1) | 0 (0.0) | 0 |
| B-2. I refer patients who have questions about fertility to an infertility specialist or reproductive endocrinologist. | 52 (34.0) | 61 (39.9) | 32 (20.9) | 7 (4.6) | 1 (0.7) | 0 |
| B-3. I discuss the impact of cancer treatment on future fertility with my cancer patients. | 75 (49.0) | 61 (39.9) | 15 (9.8) | 2 (1.3) | 0 (0.0) | 0 |
| B-4. I discuss fertility issues with patients regardless of their insurance status. | 70 (46.1) | 53 (34.9) | 20 (13.2) | 8 (5.3) | 1 (0.7) | 1 |
| B-5. I discuss fertility issues with patients regardless of pathologic staging of cancer. | 47 (30.7) | 56 (36.6) | 35 (22.9) | 14 (9.2) | 1 (0.7) | 0 |
| B-6. Someone else within my practice discusses fertility preservation with my patients. | 7 (4.6) | 21 (13.8) | 25 (16.4) | 52 (34.2) | 47 (30.9) | 1 |
| B-7. I provide my patients with educational material about fertility preservation. | 9 (5.9) | 17 (11.1) | 16 (10.5) | 72 (47.1) | 39 (25.5) | 0 |
| B-8. Are you comfortable discussing fertility preservation with your patients? | 51 (33.3) | 54 (35.3) | 31 (20.3) | 17 (11.1) | 0 (0.0) | 0 |
| B-9. In the case of fertility preservation counseling, if the patient is unable to make a decision, re-consultation helps arrive at a reasonable decision. | 29 (19.0) | 66 (43.1) | 46 (30.1) | 12 (7.8) | 0 (0.0) | 0 |

**Supplementary Table 3.** Barriers to discussing fertility preservation.

|  | **Always** | **Often** | **Sometimes** | **Rarely** | **Never** | **No opinion** |
| --- | --- | --- | --- | --- | --- | --- |
|  | **n (%)** | **n (%)** | **n (%)** | **n (%)** | **n (%)** | **n** |
| C-1. Breast cancer treatment is delayed owing to preservation of fertility. | 9 (5.9) | 38 (25.0) | 65 (42.8) | 37 (24.3) | 3 (2.0) | 1 |
| C-2. A patient does not want to discuss fertility preservation. | 0 (0.0) | 17 (11.2) | 47 (30.9) | 74 (48.7) | 14 (9.2) | 1 |
| C-3. The patient cannot make a decision after consultation. | 0 (0.0) | 44 (29.3) | 57 (38.0) | 45 (30.0) | 4 (2.7) | 3 |
| C-4. There is no place to refer my patients for fertility preservation. | 7 (4.6) | 28 (18.5) | 42 (27.8) | 47 (31.1) | 27 (17.9) | 2 |
| C-5. There is no person to whom I can refer my patients for fertility preservation. | 16 (10.7) | 32 (21.5) | 34 (22.8) | 37 (24.8) | 30 (20.1) | 4 |
| C-6. There are no obstetricians and gynecologists to request fertility preservation procedures. | 12 (7.9) | 21 (13.8) | 21 (13.8) | 42 (27.6) | 56 (36.8) | 1 |
| C-7. Time constraints affect my ability to discuss fertility preservation. | 14 (9.2) | 66 (43.4) | 41 (27.0) | 24 (15.8) | 7 (4.6) | 1 |

**Supplementary Table 4.** Fertility preservation attitudes.

|  | **Strongly agree** | **Agree** | **Neutral** | **Disagree** | **Strongly disagree** | **No opinion** |
| --- | --- | --- | --- | --- | --- | --- |
|  | **n (%)** | **n (%)** | **n (%)** | **n (%)** | **n (%)** | **n** |
| D-1. Patients with a poor prognosis should not pursue fertility preservation | 13 (8.6) | 40 (26.3) | 45 (29.6) | 49 (32.2) | 5 (3.3) | 1 |
| D-2. Fertility preservation is a high priority for me to discuss with my newly diagnosed cancer patients of childhood bearing age. | 22 (14.6) | 66 (43.7) | 46 (30.5) | 17 (11.3) | 0 (0.0) | 2 |
| D-3. Some patients fear passing on a hereditary cancer to a biological child. | 35 (23.0) | 87 (57.2) | 21 (13.8) | 8 (5.3) | 1 (0.7) | 1 |
| D-4. Some patients with certain cancer (e.g. hereditary breast and ovarian cancer) should be informed about preimplantation genetic diagnosis. | 16 (10.6) | 68 (45.0) | 49 (32.5) | 14 (9.3) | 4 (2.6) | 2 |
| D-5. Treating the primary cancer is more important than fertility preservation. | 26 (17.3) | 85 (56.7) | 26 (17.3) | 11 (7.3) | 2 (1.3) | 3 |

**Supplementary Table 5.** Fertility preservation perceptions.

|  | **Strongly agree** | **Agree** | **Neutral** | **Disagree** | **Strongly disagree** | **No opinion** |
| --- | --- | --- | --- | --- | --- | --- |
|  | **n (%)** | **n (%)** | **n (%)** | **n (%)** | **n (%)** | **n** |
| E-1. Patients ask me about the effects of cancer treatment on their fertility. | 6 (4.0) | 93 (61.6) | 34 (22.5) | 17 (11.2) | 1 (0.7) | 2 |
| E-2. Patients of lower socioeconomic status are less concerned with future fertility. | 11 (7.3) | 32 (21.2) | 54 (35.8) | 50 (33.1) | 4 (2.6) | 2 |
| E-3. Female patients are more concerned about fertility preservation than male patients. | 23 (15.4) | 82 (55.0) | 35 (23.5) | 9 (6.0) | 0 (0.0) | 4 |

**
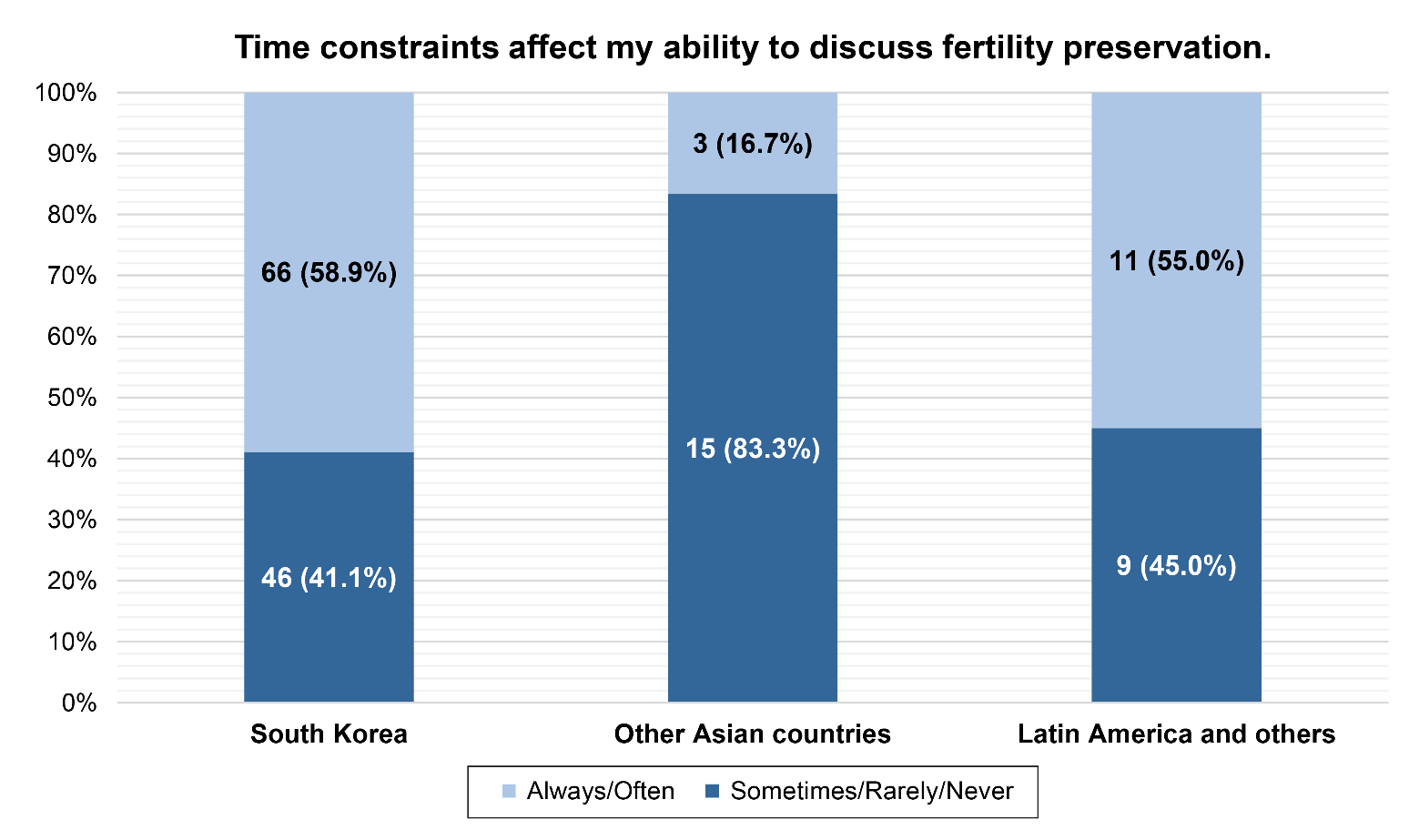
**

**Supplementary Figure 1.** Response to time constraints based on nationality.
